# Mid-term results of bilateral synchronous total hip arthroplasty for bony ankylosis in patients with ankylosing spondylitis

**DOI:** 10.1186/s13018-021-02258-z

**Published:** 2021-02-02

**Authors:** Lei Han, Renfu Quan, Zhenle Pei, Guoping Cao, Yungen Hu, Jingjing Liu

**Affiliations:** 1 Department of Orthopaedics Institute, Xiaoshan Traditional Chinese Medical Hospital, No. 152 Yucai Road, XiaoShan District, Hangzhou, 311201 Zhejiang Province China; 2 Department of Medical Center of Fudan University, No. 305 Fenglin Road, XuHui District, Shanghai City, 200433 Shanghai China; 3 Department of Rheumatology and Immunology, The Third Affiliated Hospital of Zhejiang Chinese Medical University, No. 219 Moganshan Road, Xihu District, Hangzhou, 310005 Zhejiang Province China

**Keywords:** Arthroplasty, Replacement, Hip, Spondylitis, Ankylosing spondylitis

## Abstract

**Background:**

Total hip arthroplasty (THA) for bony ankylosis is technically challenging in patients with ankylosing spondylitis (AS). This study aimed to determine the mid-term results of bilateral synchronous THA for bony ankylosis in patients with AS.

**Methods:**

Nineteen cases of bony ankylosis in patients with AS who received bilateral synchronous THA were included in this study (17 males and 2 females, mean age 49.2 years). Disease duration was 5–38 years (mean 18 years and 6 months). All patients received cementless THA. Intraoperative blood loss, visual analog scale (VAS) score, and complications were assessed. Harris hip scores evaluated the clinical effect.

**Results:**

Patients were followed up for 62–98 months (mean 82.5 months). VAS score decreased from 7.42 ± 0.92 to 2.42 ± 0.83, Harris hip score improved from 21.8 ± 7.2 to 80.3 ± 6.5, and the flexion-extension range of the hip improved from 0 to 142.3 ± 6.2°. One patient with septum bronchiale had a fracture intraoperatively and was treated with wire strapping. One patient had a traction injury of the femoral nerve postoperatively and recovered 1 year after the operation. Loosening and subsidence were not observed in all patients. Heterotopic bone formation was noted in 3 patients. No complications such as joint dislocation, acute infection, and deep vein thrombosis were found.

**Conclusion:**

Bilateral synchronous THA was effective for bony ankylosis of the hip in patients with AS because it improved patients’ quality of life and had satisfactory mid-term outcomes.

## Background

Ankylosing spondylitis (AS) is a chronic progressive autoimmune disease involving medial axis joints. It mainly includes the sacroiliac joint, spinal process, and soft tissues beside the spine and could cause spinal deformity and hip ankylosis [1]. Approximately 25 to 50% of patients with AS have hip involvement, and 50 to 90% of hip involvement is bilateral [2].

Recently, several studies within the clinical setting on the application of total hip arthroplasty (THA) for the treatment of hip ankylosis in AS patients have been reported. THA has been shown to be the most effective treatment method for hip joint ankylosis in patients with AS [3, 4]. Moreover, THA could help AS patients with osseous ankylosis by reconstructing the anatomical structure of the hip joint, restoring hip joint function, and thus significantly improving their survival and quality of life.

Hip joint ankylosis could influence the extent of surgical exposure [5]. Contracture of muscle tissue and heterotopic ossification results in further trauma and difficulties during surgery, thereby limiting the choice of surgical approach [6]. In addition, the patient’s mobility is limited after unilateral hip arthroplasty. In cases with bilateral involvement, the subsequent surgery could affect joint function recovery and the second administration of anesthesia could increase the risk of complications.

In this study, we retrospectively analyzed the data of AS patients with hip ankylosis treated by bilateral synchronous THA in our hospital, and we evaluated hip joint function and postoperative complications. The purpose of this study was to discuss the surgical sequence of spinal osteotomy and hip replacement and determine the mid-term results of bilateral synchronous THA for bony ankylosis in patients with AS.

## Methods

All study participants provided informed consent. From January 2008 to January 2013, 19 patients (38 hips) with AS were treated with bilateral THA (17 males and 2 females). Patient ages ranged from 41 to 68 years (mean age 49.2 years), and the disease duration was 5–38 years (mean 18 years and 6 months). All patients had different degrees of ankylosis and dysfunction of the hip; 12 patients completely lost their self-care ability, 3 needed a walking device, and 4 required a walking stick. Ankylosis of the hip was defined by physical examination as a total loss of hip motion. Average angle of hip flexion deformity was 17.8° (fourteen hips 20–30°, sixteen hips 10–20°, eight hips < 10°). Twelve patients had difficulty with horizontal vision due to severe spinal kyphosis and hip flexion. The preoperative visual analog scale (VAS) [7] score was 7.42 ± 0.92 and Harris hip score [8] was 21.8 ± 7.2. Bilateral total hip replacement was performed simultaneously. Eight patients first completed the spinal osteotomy. All patients received uncemented THAs: ceramic on polyethylene (friction interface) was used in 3 patients (6 hips) and ceramic on ceramic in 16 patients (32 hips). Zimmer, Link, and Stryker prostheses were used in 10, 7, and 2 patients, respectively.

### Perioperative preparation

Risk assessment was performed preoperatively according to the American Standards Association to understand the patient’s surgical tolerance. The general condition and nutritional status, cardiopulmonary function, erythrocyte sedimentation rate, c-reactive protein, X-ray, bone density, muscle strength, and the muscle and surrounding soft tissue of the hips were evaluated; the anesthesia method, prosthesis type, and operation sequence were determined accordingly. Measurements were performed using a special template based on the patient’s hip X-ray to determine the size, type, and position of the implant during surgery. Immunosuppressive drugs were discontinued 2 weeks before the surgery, and anti-inflammatory and analgesic drugs 1 week before the surgery to reduce the incidence of postoperative gastrointestinal bleeding and infection. Prophylactic antibiotics were administered 30 min before the surgery.

### Surgical methods

#### Surgical approach and exposure

Bilateral sequential operations were performed under general anesthesia under the guidance of a fiberoptic bronchoscope. The surgical areas were disinfected accordingly, the patient was placed in the lateral position, and the incision was via the posterolateral approach to fully expose the hip joint and release the soft tissue of the contracture.

#### Osteotomy

**Fig. 1:**
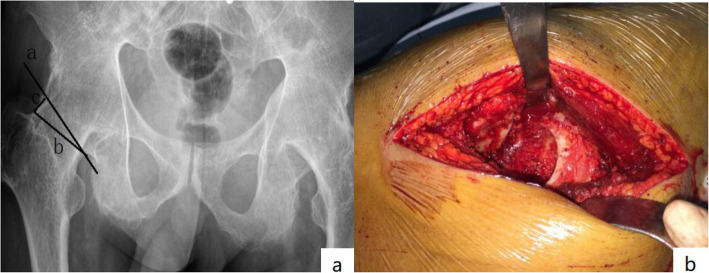
**a** Delineation of two-step system. Osteotomy perpendicular to the femoral neck 10 mm from the posterior wall of acetabulum (a). Next, wedge osteotomy was performed (b). The bottom edge of wedge osteotomy is 5 mm (c). **b** Homann hooks were placed in front and rear of the femoral neck, and osteotomy was performed at the junction of the femoral neck and acetabulum

Osteotomy of the femoral neck was performed twice [5]. First, the osteotomy was performed perpendicular to the femoral neck at 10 mm from the posterior acetabular wall. Second, wedge osteotomy was performed; the base of the wedge osteotomy was 5 mm (Fig. 1a). During osteotomy, careful attention should be paid to the junction of the femoral neck and acetabulum. Homann hooks were placed in front and at the back of the femoral neck to protect the femoral artery and the adjacent sciatic nerve (Fig. 1b). Osteotomy was near the acetabulum outer edge (15° front lean angle) to prevent posterior acetabular defects. In cases where the femoral neck was not completely cut off, fracture depth was gradually increased using a pendulum saw and bone knife under direct vision until the hip joint fusion statement was cut off.

#### Acetabular formation and acetabular prosthesis placement

Although the hip had bony fusion, there remained incomplete ossifying cartilage at the location of the original joint plane. In some instances, intraoperative radiographs were taken to identify the original joint plane, which proved useful. The abduction and forward tilt of the acetabulum were adjusted according to the spinal and pelvic deformities. The position of the acetabulum could be identified based on the iliac ischium, iliac pubic branch, and the lower margin of the iliac crest. Moreover, osteoporosis is common in AS patients. In cases with acetabular defects due to acetabular hole, the femoral neck that was removed could be put at the bottom of the acetabulum after dressing or bone graft could be added to reconstruct the bottom of the acetabulum.

#### Organizational slack

**Fig. 2:**
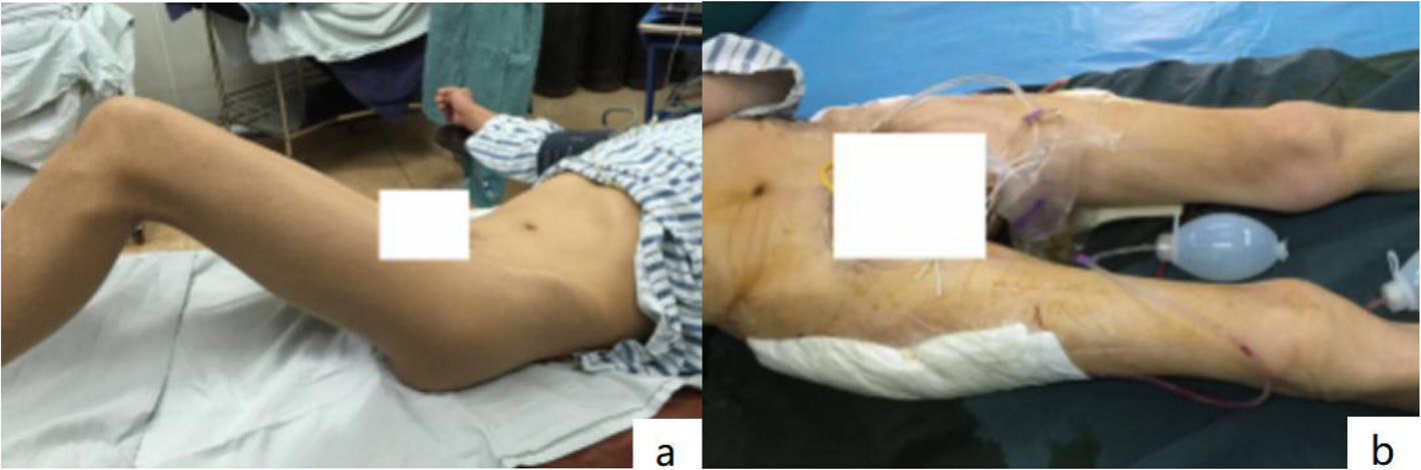
**a** The perioperative photo showed that the patient flexion deformity bilateral hips were over 45°. **b** The postoperative photo showed that the hip straightening was achieved immediately

During hip arthroplasty, the anterior soft tissues of the joint were released. The released tissues included iliopsoas, sartorius, and rectus femoris tendons and sometimes adductor tendons. After completing the operation on one side, abduction and rotation of the contralateral lower limbs were avoided to prevent joint dislocation. For those with mild hip flexion, hip straightening was achieved immediately after the release (Fig. 2a, b). Soft tissue releases were performed in 11 patients (22 hips), including 6 (12 hips) adductor releases and 5 (10 hips) iliopsoas releases.

### Postoperative treatment

Postoperative negative pressure drainage was performed for 24–48 h, and broad-spectrum antibiotics were routinely administered to prevent infection. For most patients with ankylosing spondylitis, taking hormones for a long time results in low immunity, long-term bed, or the potential-infection risk of lung and urinary system. Therefore, we extend the use of antibiotics. Occurrence of wound, swelling, and peripheral blood circulation were closely monitored, and low-molecular-heparin calcium or rivaroxaban was administered to prevent deep vein thrombosis during the perioperative period. For those who were taking hormones for the long-term, hormone therapy was allowed during the perioperative period. Oral indomethacin 25 mg/day was administered postoperatively to prevent heterotopic ossification. Isometric contraction of the lower limb muscles was performed 1 day postoperatively. Standing exercise began on day 3 after the operation at the bedside, and further exercise was performed with the help of double crutches at approximately 1 week postoperatively. For those with severe osteoporosis, weight-bearing exercises were delayed, and postoperative anti-osteoporosis treatment was continued.

### Follow-up and evaluation of efficacy

The patients were followed up at 1, 3, and 6 months and once a year thereafter for the clinical evaluation of their hip function and to assess their X-ray films. The patients’ X-ray films were sent to the examiners who were unable to come to the hospital, and the examiners completed the functional evaluation by telephone.

### Radiographic assessment

#### Acetabular lateral stability assessment

According to Kawamura [9], the assessment criteria for acetabular prosthesis loosening are as follows: stable fixation, no displacement of acetabular prosthesis, and no line of illumination; stable fiber fixation, no displacement of acetabular prosthesis, and presence of light line < 1 mm; suspected loosening, no displacement of acetabular prosthesis, and presence of progressive, discontinuous light line > 2 mm during follow-up; and definite loosening, displacement of acetabular prosthesis, or the occurrence of > 2-mm continuous bright band or screw fracture.

#### Joint prosthesis stability assessment

Prosthesis fixation was evaluated based on a previous study [10]; the following were assessed: bone growth stability (i.e., no subsidence, little or no hardening line formation of the prosthesis, and stability of most of the bone-prosthesis interface), fiber fixation stability (i.e., formation of a continuous bright band of no more than 1 mm around the prosthesis parallel to the prosthesis handle, but no progressive subsidence and displacement occurs), and prosthesis instability (i.e., progressive subsidence (≥2 mm) with new (varus or porous) surface separation or prosthesis fracture occurs).

#### Heterotopic ossification assessment

The Brooker classification [11] was adopted for the imaging evaluation of heterotopic ossification: grade I, 1 or 2 ossification areas with a diameter < 1 cm; grade II, isolated ossification or ossification at the end of the femur or the margin of the acetabulum, covering less than half the distance between the femur and the hip; grade III, less than half the distance between the femur and the hip bone without bone bridge formation; and grade IV, bridge formation between the femur and the hip bone. Data collection and evaluation were performed by independent examiners.

## Results

### General conditions of the operation

The operation time was 140–240 min (mean 180.8 min). The total amount of blood loss was 1080–1880 ml (mean 1262.5 ml). The intraoperative autologous return was 500–750 ml (mean 650.6 ml). The amount of postoperative allogeneic blood transfused was 500–1000 ml (mean 725.2 ml).

### Clinical efficacy evaluation

**Fig. 3:**
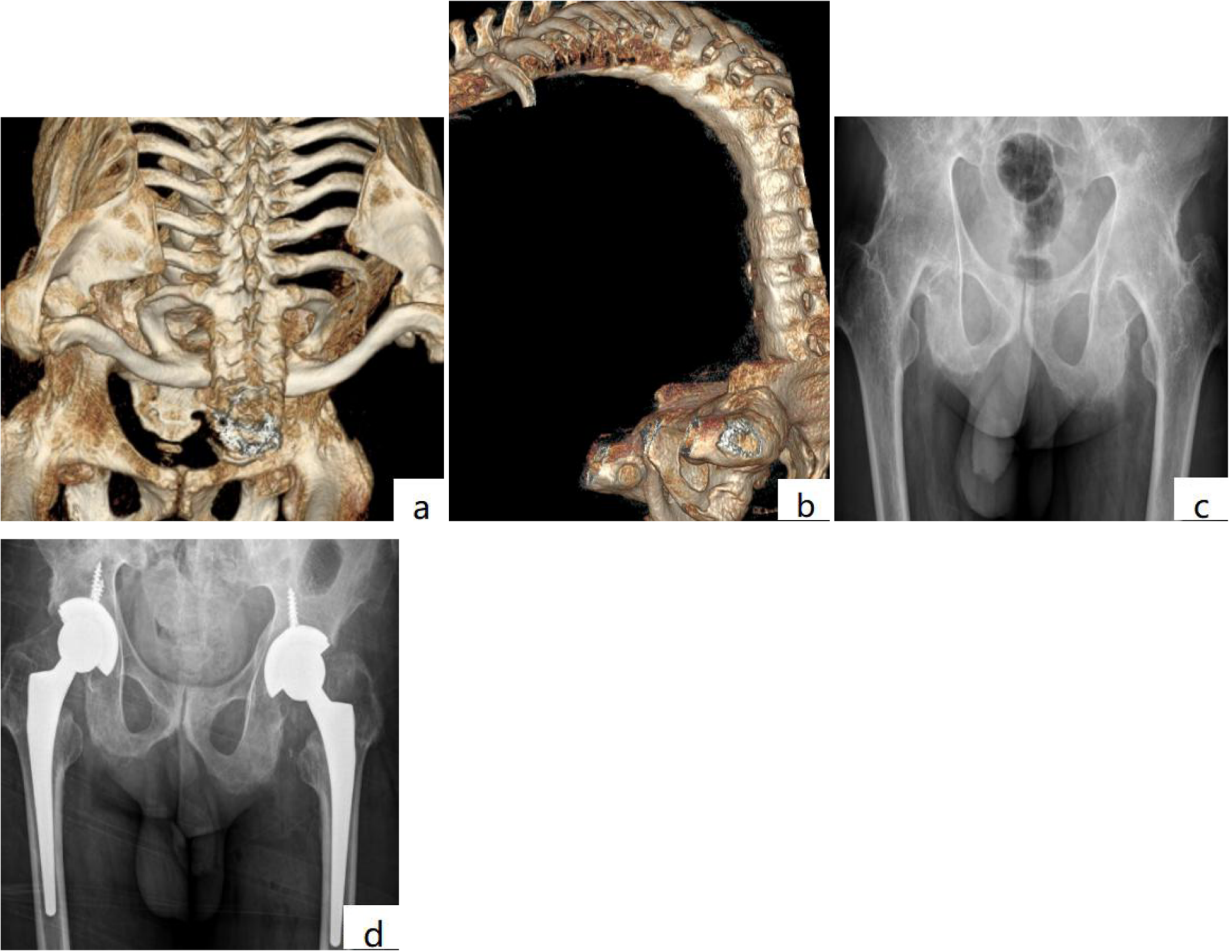
**a**, **b** Three-dimensional reconstruction showing AS with hip flexion ankylosis and kyphosis deformity. **c** The radiographs showing bilateral bony ankylosis with 0° range of motion. **d** Postoperative radiograph of the patient 5 years after cementless THA

The patients were followed up for 62–98 months (mean 82.5 months). The VAS score decreased from 7.42 ± 0.92 (preoperative) to 2.42 ± 0.83 (postoperative) points (*t* = 25.83, *P* < 0.001). Joint function based on Harris hip score improved (from 21.8 ± 7.2 to 80.3 ± 6.5) (*t* = − 73.50, *P* < 0.001), among which 3 were excellent cases (6 hips) and 12 were good cases (24 Hip), 3 were medium cases (6 hips), and the excellent and good rate was 78.9%. The range of hip motion (sum of flexion, extension, adduction, abduction, internal rotation, external rotation, etc.) increased from 0 to 142.3 ± 6.2° (*t* = − 71.42, *P* < 0.001). Four patients could walk with a single crutch, and the rest could walk unassisted. Six patients underwent a second treatment with spinal osteotomy (Fig. 3a, b). The long-term contracture of the hip changes the biomechanical properties of the lower limbs, leading to secondary injury of the knee. Two patients had knee pain postoperatively, and the pain was relieved after total knee replacement.

### Imaging evaluation of stability

Postoperative X-ray images showed that both the acetabulum and the femoral stem prosthesis had a good biological pressure coordination effect, and the femoral stem prosthesis was fixed at a neutral position or everted position (Fig. 3c, d). Acetabulum abduct angles were 40.2 ± 10.2° and the anteversion was 13.5 ± 5.8°. Radiographic images showed extensive bone growth on the surface of the prosthesis 6 months after the operation. The femoral stem prosthesis and bone interface were assessed as bone fixation according to the standards of Engh et al. [10], and acetabular prosthesis was assessed as stable according to the standards of Kawamura [9] with no visible lines. Moreover, X-ray showed no subsidence of the femoral prosthesis at 1 year follow-up postoperation.

Heterotopic ossification occurred in 3 cases (6 hips). According to Brooker classification, there were 2 cases (4 hips) in grade I and 1 cases (2 hips) in grade II.

### Complications

Intraoperative femoral neck osteotomy was successfully completed. Two patients developed hypoproteinemia and occurred incision seepage at postoperative days 2 and 3. After multiple dressing changes and albumin supplementation, the incision was healed. A patient with a flexion deformity > 60° had femoral nerve traction injury after surgery but recovered a year thereafter. One patient had femoral condyle fracture; weight bearing was delayed, and bone healing was noted at 8 weeks after surgery. No complications, such as joint dislocation, acute infection, or deep vein thrombosis of the lower extremities, occurred.

## Discussion

In this study, bilateral THA was performed first in 11 patients, and 5 patients did not undergo spinal osteotomy because they could meet daily activities. In addition, 6 patients who underwent spinal osteotomy showed improvement in gait and maintained an effective range of hip joint motion.

Studies have shown that THA is the best treatment method for advanced hip bony ankylosis in patients with AS. Bilateral synchronous hip replacement for hip ankylosis in patients with AS shortens the length of hospital stays and avoids the risks of multiple operations and anesthesia. Moreover, rehabilitation could be performed simultaneously, which in turn reduces the incidence of complications and maximizes recovery of function. The incidence of complications after bilateral THA is much lower than that after two-stage replacements [12]. However, AS is often accompanied by spine or pelvic deformity, severe osteoporosis, and tissue contracture. Thus, a bilateral simultaneous operation is extremely difficult to perform in patients with AS and has higher requirements for operation sequence, surgical operation, and postoperative rehabilitation.

Currently, there is no definite surgical sequence for kyphosis or hip ankylosis in patients with AS [13]. The operation sequence is not important in cases with mild flexion deformity of the hip joint. For those with severe flexion deformity of the hip, THA could be performed first as the hip joint could partly compensate for the kyphosis and the placement of the body position can make it easier to determine the kyphosis angle, which is beneficial for the further precise spinal osteotomy [5, 14]. In addition, shear strength of the spine in the standing position is minimal, and the incidence of spondylolisthesis and kyphosis recurrence is reduced. Other scholars believe that the first spinal surgery could affect the range of hip joint movement, avoid the wrong direction of prosthesis placement, and reduce the incidence of hip dislocation [15]. Nevertheless, the best surgical plan is that which considers both the spine and the hip joints. Moreover, surgery should follow the following principles:

1. The spine surgeon determines the direction of the trunk axis that is changed by spinal osteotomy, and the joint surgeon determines the range of motion of the hip after THA; the two coordinate with each other to match the effective range of motion of the hip to the new trunk axis.
2. Based on the information in principle 1, THA is performed first followed by spinal osteotomy. The acetabular prosthesis should be placed according to the functional position and conforming to the direction of the trunk axis that was changed by spinal osteotomy.
3. Postoperative exercise can not only be satisfied with being able to sit, but also for different degrees of squatting, to ensure that there is enough space for the spinal osteotomy.
4. After 3 months of stable hip function, spinal osteotomy orthopedic surgery should be performed. Hip range of motion should be evaluated again before surgery to determine the extent of spinal osteotomy.

Accurate placement of the acetabular component is one of the difficulties in AS patients with hip bony ankylosis. Generally, the foveal soft tissue can be identified in the location of the original joint plane [5]. The pelvis has three bony landmarks that determine the guide and accurate placement of the acetabular cup [16]. In this study, these landmarks were used during the operation and the postoperative acetabular cup position was satisfactory and no dislocation occurred. In addition, intraoperative fluoroscopy could be useful for identifying the exact location of the acetabulum [5, 17]. Since kyphosis is often present in AS, pelvic posterior tilt compensation mechanism should be considered in the placement of acetabulum during the operation. If the acetabulum is tilted forward and the abduction angle is normal, the pelvis should be tilted backward when the patient is standing to increase the anteversion and abduction angles of the acetabular prosthesis. Hence, the anteversion and abduction angles of the acetabulum should be reduced during the operation to offset the anteversion and abduction angles resulting from pelvic recline; however, pelvic recline decreases or disappears after spinal orthopedic surgery [18, 19], and the original suitable acetabular abduction and anteversion angles are extremely small, which could in turn result in complications such as frontal impact and limited hip flexion. Subsequent spinal osteotomy reduces partial motion of hip flexion after THA. The acetabulum should be placed according to the functional position of the trunk after spinal orthosis. For the functional exercise after THA, hip flexion range of motion should be monitored closely, and excessive extension should be avoided. Correction surgery for kyphosis could make up for the lack of straightening.

Osteoporosis not only increases the difficulty of surgery but also affects the stability of the prosthesis. Although cemented prosthesis has good early stability, it is more difficult for revision. Nevertheless, restoring joint function and resumption of weight bearing could improve osteoporosis. Thus, if the bone condition permits, the cementless prothesis should be selected as far as possible for revision THA later. During follow-up, no patients in this study experienced loosening of the prosthesis due to osteoporosis.

Moreover, because of the gradual recovery of joint function, the bone density of the acetabulum and femur gradually increased, and the postoperative biological bone ingrowth was satisfactory. For the chimney-shaped medullary cavity, we used a tapered femoral stem, and a sagittal three-point fixation in the chimney-shaped wide medullary cavity was achieved using a longer shank, which is beneficial to the initial stability of the prosthesis, the double-sided tapered design can obtain a secondary sinking to make the handle body firmer and facilitate the growth of the proximal bone.

Heterotopic ossification is an important factor affecting joint mobility in patients with AS after total hip arthroplasty [20, 21]. The incidence of heterotopic ossification in patients with AS after THA treatment is as high as 40–76% [22]. Severity of heterotopic ossification complications varies, and severe cases are often prone to joint function alterations and can affect postoperative outcomes. Intraoperative osteotomy, organization of hematoma, soft tissue injury, and residual bone debris could influence the formation of heterotopic ossification [23]. In this study, repeated washing with a large amount of normal saline was performed during operation to completely remove the residual bone and minimize the damage to soft tissues around the hip. After routine oral administration of indomethacin (25 mg/day) for 15 days, heterotopic ossification was effectively reduced postoperatively. Mild heterotopic ossification was noted in 3 patients (6 hips) (Brooke I and II), and hip activity was not affected. The postoperative irradiation is effective in preventing the occurrence of heterotopic ossification after THA in patients with AS [24].

Most of the patients remain in bed for a long time; thus, their muscles could undergo severe atrophy. Hence, a practical rehabilitation plan is needed after the operation that includes passive and active range of motion, physical exercise, muscle and joint coordination exercise, and normal gait practice. Passive activity could increase the stretchability of the soft tissue around the joint, thereby increasing the coordination of movements in active range of motion. Therefore, early postoperative rehabilitation exercises require both passive and active range of motion but within a safe range. However, for patients with severe flexion deformity before surgery, excessive passive extension of the hip joint must be avoided to prevent nerve and blood vessel traction injury. This study of patients with mild hip flexion obtained straight hips immediately postoperation. The hip can be stretched straight by functional exercise after 3 days for the seriously flexed hips.

Although the short-term outcome after THA in patients with AS is satisfactory, the mid- and long-term results vary [25]. In the early stage, because of osteoporosis, loosening and displacement of the prosthesis after surgery are highly possible. The longer the course of AS, the more severe the hip joint involvement and the slower the recovery after surgery; moreover, the recovery was relatively poor. In addition, the longer the course of AS, the more severe the deformity of the hip joint, the ankylosis, the atrophy of the muscle, and even the fibrosis, which could in turn result in operation difficulties, operation trauma, and prolonged operation time and could directly affect recovery speed and postoperative satisfaction. In this study, we found that the bilateral hip joints improved, and early rehabilitation exercises could be performed. Moreover, postoperative synchronous rehabilitation exercises were performed to improve operative outcomes.

## Conclusion

THA is an effective treatment method for hip ankylosis in patients with AS and thus could improve the hip joint function and the quality of life. Bilateral synchronous THA shortens the time of hospitalization, reduces the risk of anesthesia, and relieves patients’ cost burden. The mid-term results of bilateral synchronous THA are satisfactory. This study lacks a control group and needs a multi-center, large sample study to confirm its exact effect.

## Data Availability

This data will not be shared, because in recent years, although many scholars have explored this in various aspects, its pathological mechanism remains unclear and there are no standard diagnostic criteria. In order to determine the effective method for preventing and treating this disease, it is necessary to proceed with more large-scale and clinical studies.

## Abbreviations

AS: Ankylosing spondylitis
THA: Total hip arthroplasty
VAS: Visual analog scale
